# Recurrent thrombotic events in a patient with endometrial cancer: A case report of Trousseau syndrome

**DOI:** 10.1097/MD.0000000000042503

**Published:** 2025-05-30

**Authors:** Xin-xin Yan, Qian-ying Zhao, Xiu Zheng, Zhi-rong Wan

**Affiliations:** a Department of Geriatrics 1, Aerospace Center Hospital, Peking University Aerospace School of Clinical Medicine, Beijing, China.

**Keywords:** anticoagulation, case report, endometrial cancer, thrombotic events, Trousseau syndrome

## Abstract

**Rationale:**

Trousseau syndrome is a well-recognized paraneoplastic syndrome that manifests as thrombosis in patients with malignancies. It is particularly associated with mucin-producing adenocarcinomas and is known to lead to significant morbidity and mortality due to its thrombotic complications. The underlying mechanisms involve a hypercoagulable state induced by cancer-related factors, which necessitates vigilant monitoring and management.

**Patient concerns:**

In this case, we report a 53-year-old woman with endometrial cancer who experienced recurrent thrombotic events. Her background history included breast cancer and 5-months hypertension in drug treatment.

**Diagnoses:**

Aging examination showed bilateral pulmonary artery thrombosis and multiple acute cerebral infarctions in both cerebral and cerebellar hemispheres. Laboratory examinations revealed an increased D-dimer level.

**Interventions:**

The patient was treated with rivaroxaban for anticoagulation for 1-month.

**Outcomes:**

Cerebral infarction occurred again, and the D-dimer level increased again. After adjustment to low-molecular-weight heparin treatment, the patient’s condition was stable, and no new infarction was found on follow-up brain magnetic resonance imaging.

**Lessons:**

This case underscores the complexities involved in managing thrombotic complications in patients diagnosed with Trousseau syndrome. It illustrates that thrombotic events may persist even with appropriate anticoagulant therapy, which can lead to a poor prognosis for affected patients. Nevertheless, it also emphasizes the importance of proactive monitoring and tailored management strategies, which can significantly reduce the incidence of thrombotic events and improve patient outcomes.

## 1. Introduction

Trousseau syndrome (TS), initially documented in 1865, is a clinical condition distinguished by the presence of thromboembolic incidents in individuals with concurrent malignancies. This syndrome is generally associated with grave prognosis and frequently signifies an advanced cancer stage. The underlying pathophysiology of TS is rooted in a hypercoagulable state precipitated by malignancy, which can result in both venous thromboembolism and arterial blockage. Clinical signs may include deep vein thrombosis, pulmonary embolism, and cerebral infarctions, thus necessitating heightened awareness of potential underlying malignancies in patients who exhibit unexplained thrombotic events. Recent literature underscores the correlation between TS and various types of cancer, particularly those affecting the gastrointestinal and gynecological systems, thereby highlighting the need for careful monitoring and management of thrombotic complications in these patients.^[1,2]^

## 2. Case report

A 53-year-old female patient initially presented with vaginal bleeding. An ultrasound examination revealed an endometrial thickness of 1.4 cm with heterogeneous echotexture. Histopathological and immunohistochemical analyses were consistent with poorly differentiated endometrial adenocarcinoma, raising suspicion for dedifferentiated endometrial carcinoma. Immunohistochemical results included: β-catenin (membrane +), ER (+60%), HER2 (1+), Ki-67 (+70%), P16 (+), P53 (+99%), pan-TRK (−), PD-L1 (CPS = 2) using both 2C3 and 22C3 antibodies, PMS2 (+), PR (+20%), PTEN (+), and WT1 (+). Following multidisciplinary consultation, the diagnosis was revised to carcinosarcoma or endometrial serous carcinoma. Further evaluation confirmed Stage IVB endometrial cancer with extensive metastatic disease. Her background history included 5-months hypertension in drug treatment. In 2015, she also had a breast cancer that has been under operation and adjuvant chemotherapy. After 9 years of endocrine therapy, there was no recurrence of breast cancer.

**Figure 1.**
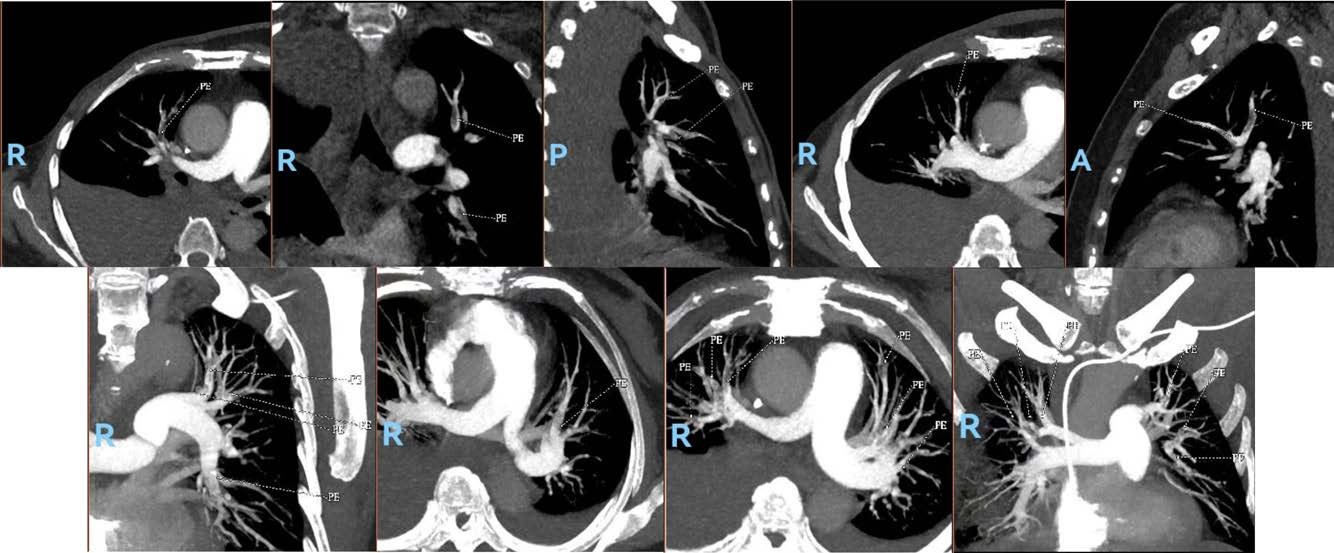
CTPA showed multiple emboli in both pulmonary arteries. CTPA = computed tomography pulmonary angiography.

**Figure 2.**
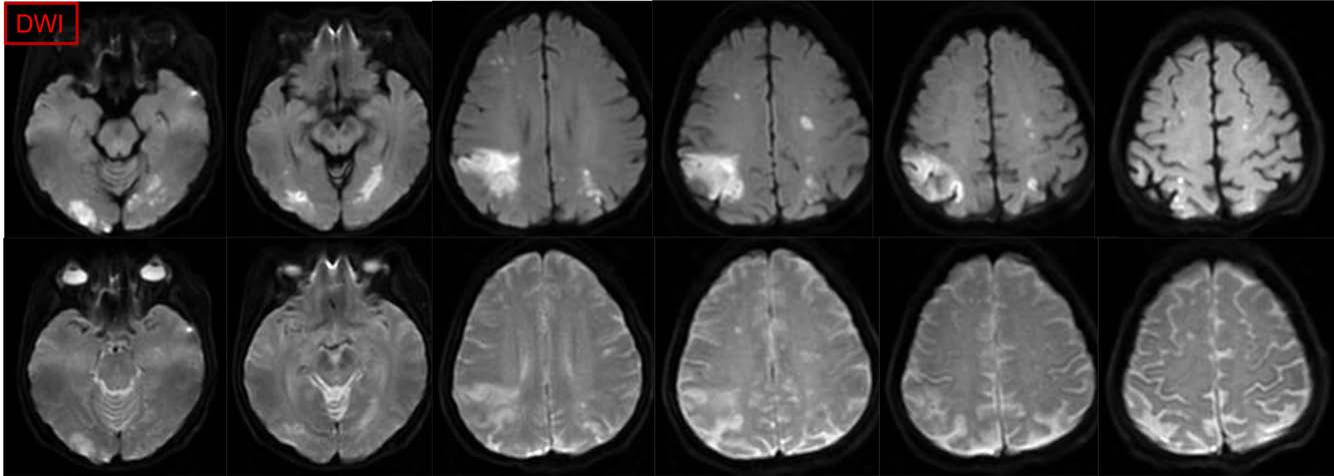
Brain MRI revealed multiple acute cerebral infarctions in bilateral cerebral and cerebellar hemispheres, especially in the right parietal lobe (DWI). DWI = diffusion weighted imaging, MRI = magnetic resonance imaging.

**Figure 3.**
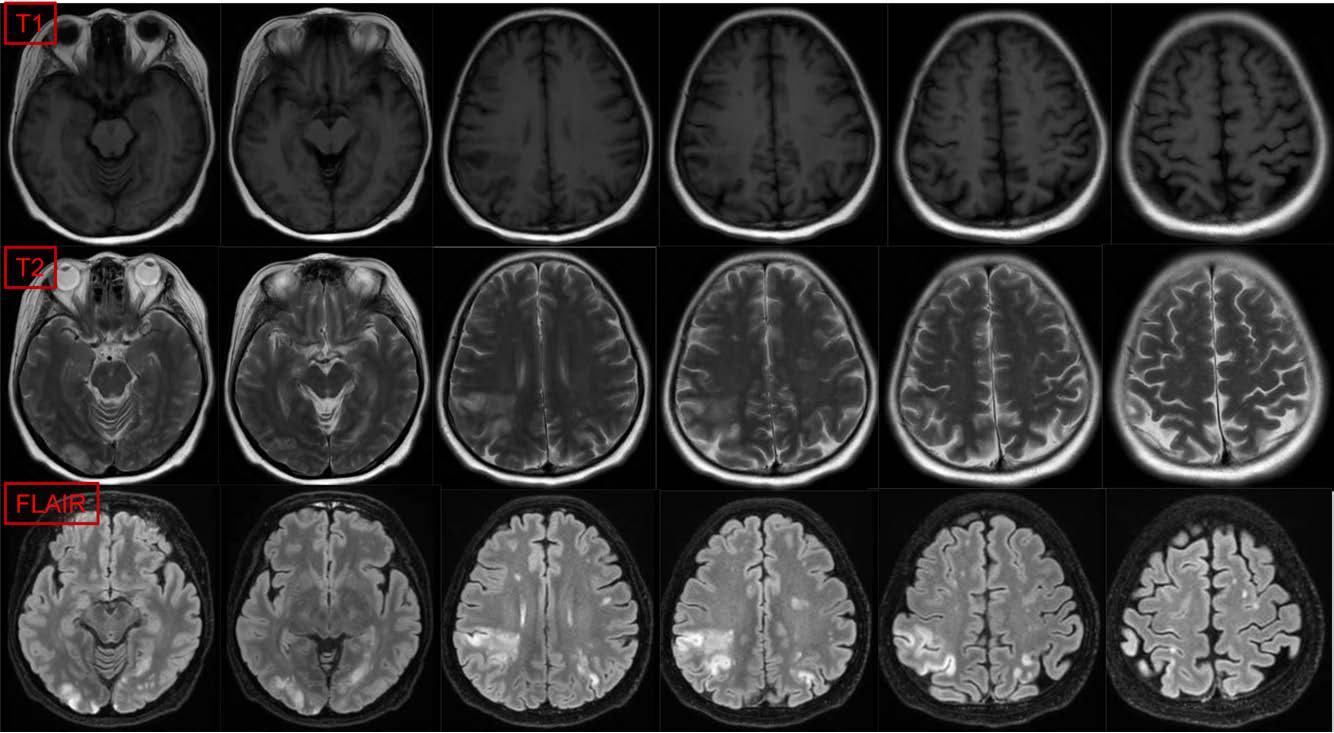
Brain MRI revealed multiple acute cerebral infarctions in bilateral cerebral and cerebellar hemispheres, especially in the right parietal lobe (T1, T2, FLAIR). MRI = magnetic resonance imaging.

**Figure 4.**
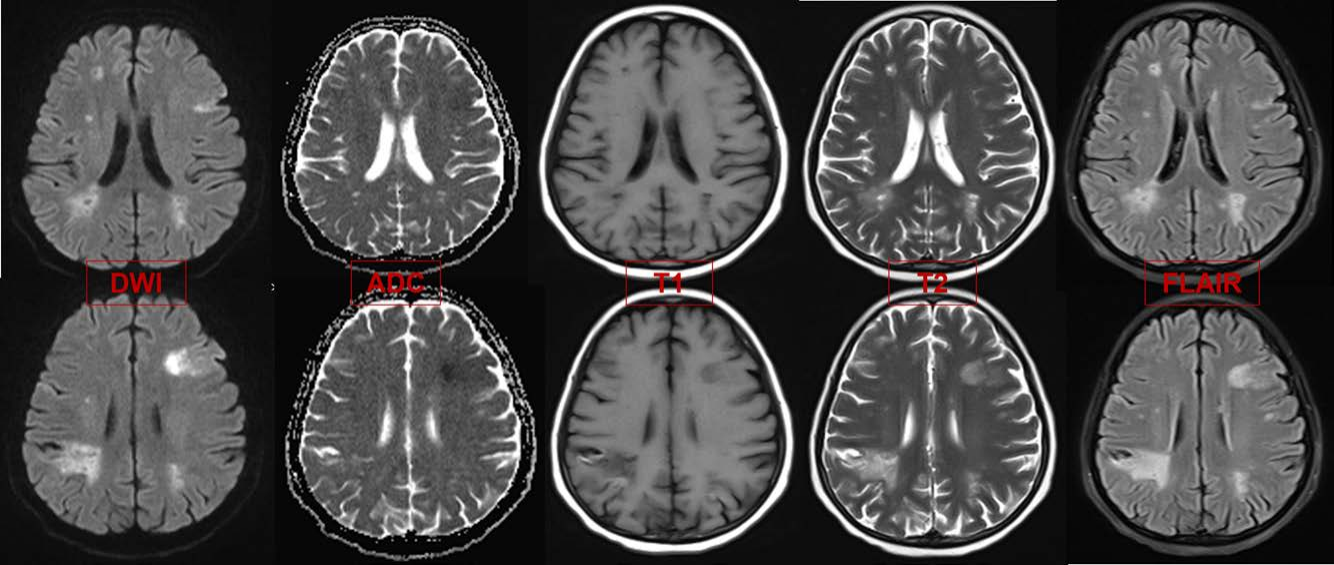
Cranial MRI showed new lesions in bilateral frontal lobe and left medial temporal lobe. MRI = magnetic resonance imaging.

The patient commenced treatment with paclitaxel liposomes, carboplatin, and bevacizumab on December 29, 2023. Following the 1st cycle, the patient developed severe vaginal bleeding, necessitating hospitalization. Neurological symptoms ensued, including dysgraphia and strabismus, along with persistent breathlessness and D-dimer level elevation. Computed tomography pulmonary angiography revealed multiple pulmonary embolisms (Fig. 1), and brain magnetic resonance imaging (MRI) confirmed bilateral cerebral and cerebellar infarction (Figs. 2 and 3). The patient underwent interventional embolization to control bleeding, followed by anticoagulant therapy initiated with enoxaparin sodium, gradually escalating to a full therapeutic dose. Posttreatment, her respiratory status improved significantly, with blood oxygen saturation returning to 98% and neurological symptoms diminishing. Owing to a partial response to chemotherapy, her treatment was continued. After discharge, she was transitioned to oral rivaroxaban (20 mg/d) but after 1 month presented with unsteady walking, and subsequent MRI revealed a new cerebral infarction (Fig. 4). The regimen was adjusted to enoxaparin sodium 4000 IU subcutaneously every 12 hours, leading to gradual alleviation of neurological symptoms and stability in her overall condition. Follow-up imaging revealed no new infarction. After 6 cycles of paclitaxel and carboplatin, the disease showed a partial response. After half a year of follow-up and monitoring (including D-dimer level, lower extremity venous thrombosis, and brain MRI examination), the patient had no further thromboembolic events and the endometrial cancer was stable.

## 3. Discussion

TS is a paraneoplastic disorder that manifests as a hypercoagulable condition linked to the presence of malignancies, resulting in repeated thrombotic occurrences. This syndrome is especially prevalent among individuals diagnosed with a range of cancers such as endometrial cancer,^[2]^ where the tumor may produce mucin, which exacerbates the hypercoagulable state.^[3,4]^ This syndrome is clinically characterized by the occurrence of venous and arterial thrombosis, which frequently complicates treatment of the associated malignancy.

In endometrial cancer, TS poses considerable clinical difficulties owing to its propensity for recurrent thrombotic incidents. Such complications may stem from various mechanisms, including nonbacterial thrombotic endocarditis, direct invasion or compression of blood vessels by the tumor, and systemic hypercoagulability. The emergence of these thrombotic events demands diligent monitoring and management approaches to alleviate the risks associated with both the cancer itself and its paraneoplastic consequences.

The “3-field sign,” observed in brain imaging studies of patients with TS, is characterized by multiple infarcts distributed across different vascular territories. This radiological hallmark aids clinicians in distinguishing TS from other stroke etiologies, and suggests an underlying malignancy when encountered. Cerebral infarctions resulting from TS are associated with poor prognosis,^[3]^ exhibiting an in-hospital mortality rate of approximately 20%, which is 3.5 times greater than that observed in the general stroke population.^[5]^ In our case of endometrial cancer, this distinct pattern underscores the importance of recognizing TS as a potential complication in patients presenting with unexplained thrombotic events.

In this case, the patient responded to low-molecular-weight heparin, but his symptoms recurred after switching to rivaroxaban, a novel oral anticoagulant, which suggests that oral anticoagulation is ineffective. It has been reported in the literature that heparin is the standard treatment for Trousseau’s syndrome-related cerebral infarction, which is superior to oral anticoagulants,^[1]^ warfarin is considered to be ineffective,^[6,7]^ and dabigatran is also reported to be ineffective in inhibiting the recurrence of Trousseau’s syndrome-related cerebral infarction.^[8]^ In this case, we realized that in addition to low molecular weight heparin playing a better role than other types of anticoagulant drugs in the treatment of TS, regular follow-up and monitoring of D-dimer, lower limb venous thrombosis and brain MRI also contributed to early detection of problems and early intervention.

## 4. Conclusion

In summary, TS poses significant diagnostic and therapeutic challenges in patients with malignancies, such as endometrial cancer. The interplay between tumor biology and coagulation abnormalities necessitates heightened awareness among clinicians to ensure timely diagnosis and appropriate management of this complex condition.^[9]^ Understanding the mechanisms underlying TS can enhance patient care by facilitating early intervention strategies aimed at reducing thromboembolic complications while effectively addressing the underlying malignancy. Heparin remains the standard treatment for patients with TS and is more effective than oral anticoagulants.

## Author contributions

**Conceptualization:** Xin-xin Yan.

**Data curation:** Qian-ying Zhao, Xiu Zheng.

**Funding acquisition:** Xin-xin Yan.

**Formal analysis:** Qian-ying Zhao.

**Software:** Xiu Zheng.

**Supervision:** Zhi-rong Wan, Xiu Zheng.

**Writing – original draft:** Xin-xin Yan, Qian-ying Zhao.

**Writing – review & editing:** Xin-xin Yan, Zhi-rong Wan.
